# Dynamics and fragmentation mechanism of (C_5_H_4_CH_3_)Pt(CH_3_)_3_ on SiO_2_ surfaces

**DOI:** 10.3762/bjnano.9.66

**Published:** 2018-02-23

**Authors:** Kaliappan Muthukumar, Harald O Jeschke, Roser Valentí

**Affiliations:** 1 Institut für Theoretische Physik, Goethe-Universität, Max-von-Laue-Straße 1, 60438 Frankfurt am Main, Germany; 2 Research Institute for Interdisciplinary Science, Okayama University, 3-1-1 Tsushima-naka, Kita-ku, Okayama 700-8530, Japan

**Keywords:** deposition, dissociation, electron beam induced deposition (EBID), focused electron beam induced deposition (FEBID), precursor, trimethyl(methylcyclopentadienyl)platinum(IV) ((CH_3_-C_5_H_4_)Pt(CH_3_)_3_)

## Abstract

The interaction of trimethyl(methylcyclopentadienyl)platinum(IV) ((C_5_H_4_CH_3_)Pt(CH_3_)_3_) molecules on fully and partially hydroxylated SiO_2_ surfaces, as well as the dynamics of this interaction were investigated using density functional theory (DFT) and finite temperature DFT-based molecular dynamics simulations. Fully and partially hydroxylated surfaces represent substrates before and after electron beam treatment and this study examines the role of electron beam pretreatment on the substrates in the initial stages of precursor dissociation and formation of Pt deposits. Our simulations show that on fully hydroxylated surfaces or untreated surfaces, the precursor molecules remain inactivated while we observe fragmentation of (C_5_H_4_CH_3_)Pt(CH_3_)_3_ on partially hydroxylated surfaces. The behavior of precursor molecules on the partially hydroxylated surfaces has been found to depend on the initial orientation of the molecule and the distribution of surface active sites. Based on the observations from the simulations and available experiments, we discuss possible dissociation channels of the precursor.

## Introduction

Nanoscale device applications require a growth of regular or specially patterned transition metal nanodeposits. Electron beam induced deposition (EBID), is a size and shape selective deposition process capable of writing low dimensional, sub-10 nm patterns on conducting and insulating substrates with tunable electronic properties [1–5]. However, the deposits obtained often contain less than 50% of metal, which is detrimental to their conductivity. The incomplete dissociation of the precursor molecules on the substrate during the deposition process leaves a significant organic residue, thus impairing the quality of the deposits [3,6]. This lowers the range of applicability of EBID for nanotechnological applications. Several postfabrication approaches (such as annealing, post-deposition annealing in O_2_, exposure to atomic hydrogen, post-deposition electron irradiation) were proposed as viable techniques [7–8], but these approaches are not completely free from reproducibility issues. Therefore, to improve the metal content and to address the nature of the organic contamination, a fundamental understanding of how the molecules behave on the substrates is necessary. This will be helpful, either to modify the existing class of precursor materials or to design a novel set of precursors, specific for electron beam deposition. To address this, we have made a series of DFT studies in which we considered fully and partially hydroxylated SiO_2_ surfaces as a representative for untreated and electron beam pretreated substrates and investigated the adsorption [9–10] and dynamics of several carbonyl precursors [11].

(C_5_H_4_CH_3_)Pt(CH_3_)_3_, in which Pt bonds directly to three methyl groups and a methylated cyclopentadienyl ring is a widely used precursor to obtain Pt deposits. Although the dissociation mechanism of (C_5_H_4_CH_3_)Pt(CH_3_)_3_ leading to the Pt deposit remains unknown, studies for a family of precursors similar to (C_5_H_4_CH_3_)Pt(CH_3_)_3_ in atomic layer deposition (ALD) conditions are available [12]. The studies in this review fairly agree that (1) the presence of surface hydroxyl groups are the source for protons that help in the evolution of H_2_, CH_4_ and H_2_O during the deposition process, and (2) the molecules dissociate or associate through a ligand exchange process [13]. Recently, examination of the behavior of this molecule on fully hydroxylated SiO_2_ surfaces has shown that the molecule remains physisorbed [14]. These static *T* = 0 K DFT computations provide insights on the bonding of (C_5_H_4_CH_3_)Pt(CH_3_)_3_ to this surface, but are limited to the adsorption behavior of the molecule. Several questions remain, such as the behavior of (C_5_H_4_CH_3_)Pt(CH_3_)_3_ on electron beam pretreated substrates, the role of temperature in the dissociation of the precursor on untreated surfaces, and the possible mechanism by which the precursor dissociates on the electron beam pretreated surfaces.

Hence, in order to extend the knowledge on the adsorption and to address the open questions in the deposition process, in this study we use DFT and finite temperature DFT-based molecular dynamics (MD) simulations and investigate the adsorption behavior of (C_5_H_4_CH_3_)Pt(CH_3_)_3_ on fully and partially hydroxylated SiO_2_ surfaces. We focus on explaining the initial reactions and the possible fragmentation pathways of the (C_5_H_4_CH_3_)Pt(CH_3_)_3_ molecule on the SiO_2_ surface, and we explain the nature of organic contamination in the deposits.

## Computational Details

DFT calculations for the substrates, (C_5_H_4_CH_3_)Pt(CH_3_)_3_ precursor molecules, and the combined substrate/precursor molecule system were performed using the projector augmented wave (PAW) method [15–16] as implemented in the Vienna ab initio simulation package (VASP-5.2.11) [17–19]. The generalized gradient approximation in the parameterization of Perdew, Burke and Ernzerhof [20] was used as approximation for the exchange and correlation functional. In addition, dispersion corrections [21] were used to simulate the long-range van der Waals interactions. All calculations were performed in the scalar relativistic approximation. A kinetic energy cut-off of 400 eV was used and all ions were fully relaxed using a conjugate gradient scheme until the forces were less than 0.01 eV/Å. In the geometry optimizations for the molecule and the surface models the Brillouin zone was sampled at the Γ point only. Spin polarization was considered for all calculations. Different spin states (i.e., in each case the two lowest possible spin states) were considered, and only the results of the ground state are reported below. The adsorption energy (*E*_A_) was defined as *E*_A_ ≡ Δ*E* = *E*_total_ − *E*_substrate_ − *E*_adsorbate_, where *E*_total_, *E*_substrate_, and *E*_adsorbate_ are the energies of the combined system (adsorbate and the slab), of the slab, and of the adsorbate molecule in the gas phase in a neutral state, respectively.

**Scheme 1:**
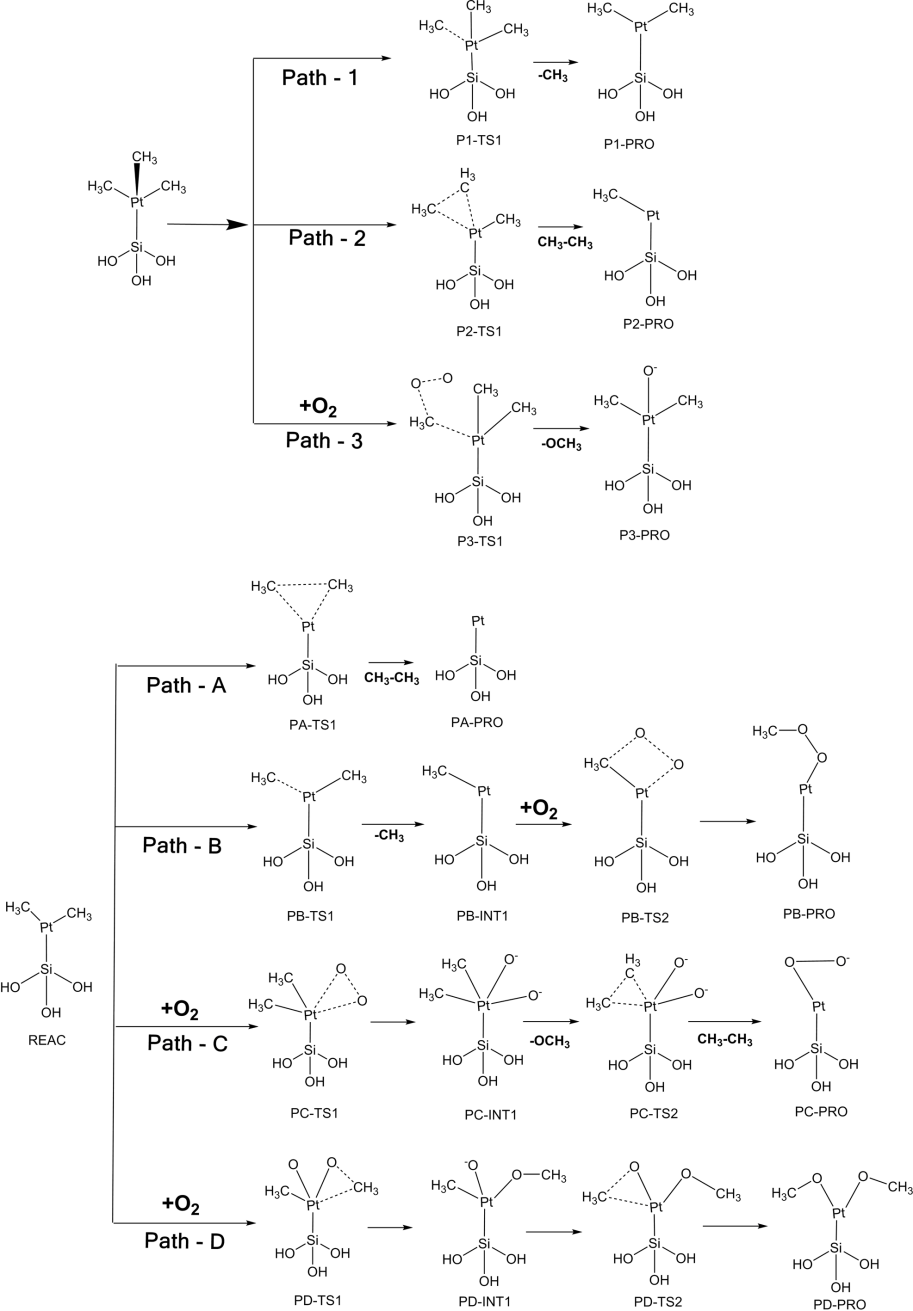
Possible reaction channels for the dissociation of (C_5_H_4_CH_3_)Pt(CH_3_)_3_ after its interaction with partially hydroxylated SiO_2_ surfaces and in an O_2_ environment. Possible pathways for the release of (top panel) a first methyl group and of (bottom panel) subsequent methyl groups.

The most stable systems were considered for studying the dynamics of the adsorbed precursor molecule. MD simulations were performed for 20 ps on a canonical ensemble at a finite temperature of *T* = 600 K using the Nose–Hoover thermostat [22]. The temperature of 600 K [23] was chosen in accordance with a typical experiment used for (C_5_H_4_CH_3_)Pt(CH_3_)_3_ deposition. The Verlet algorithm in its velocity form with a time step of Δ*t* = 1 fs was used to integrate the equations of motion. For these simulations, we have used a reduced (2 × 2 × 2) SiO_2_ supercell in order to reduce the computational time. For reaction modeling studies, all species (transition states (TS) and intermediates (INT)) in the proposed reaction paths given below in Scheme 1 were traced at PM6 (parametrized model 6) level using the Berny algorithm implemented in the Gaussian 09 software [24].

## Results and Discussion

### Adsorption of (C_5_H_4_CH_3_)Pt(CH_3_)_3_ on SiO_2_ substrates

The interaction of the precursor molecule with the substrate, in general, depends on both the orientation of the adsorbate and the adsorption site on the substrate. The interaction of (C_5_H_4_CH_3_)Pt(CH_3_)_3_ with the fully hydroxylated SiO_2_ substrate surfaces was investigated by placing the molecule with different orientations on several bonding sites and the most stable configuration is the one in which the methylcyclopentadienylring and two of the methyl groups that are directly bonded to Pt are oriented towards the substrate [14].

**Figure 1:**
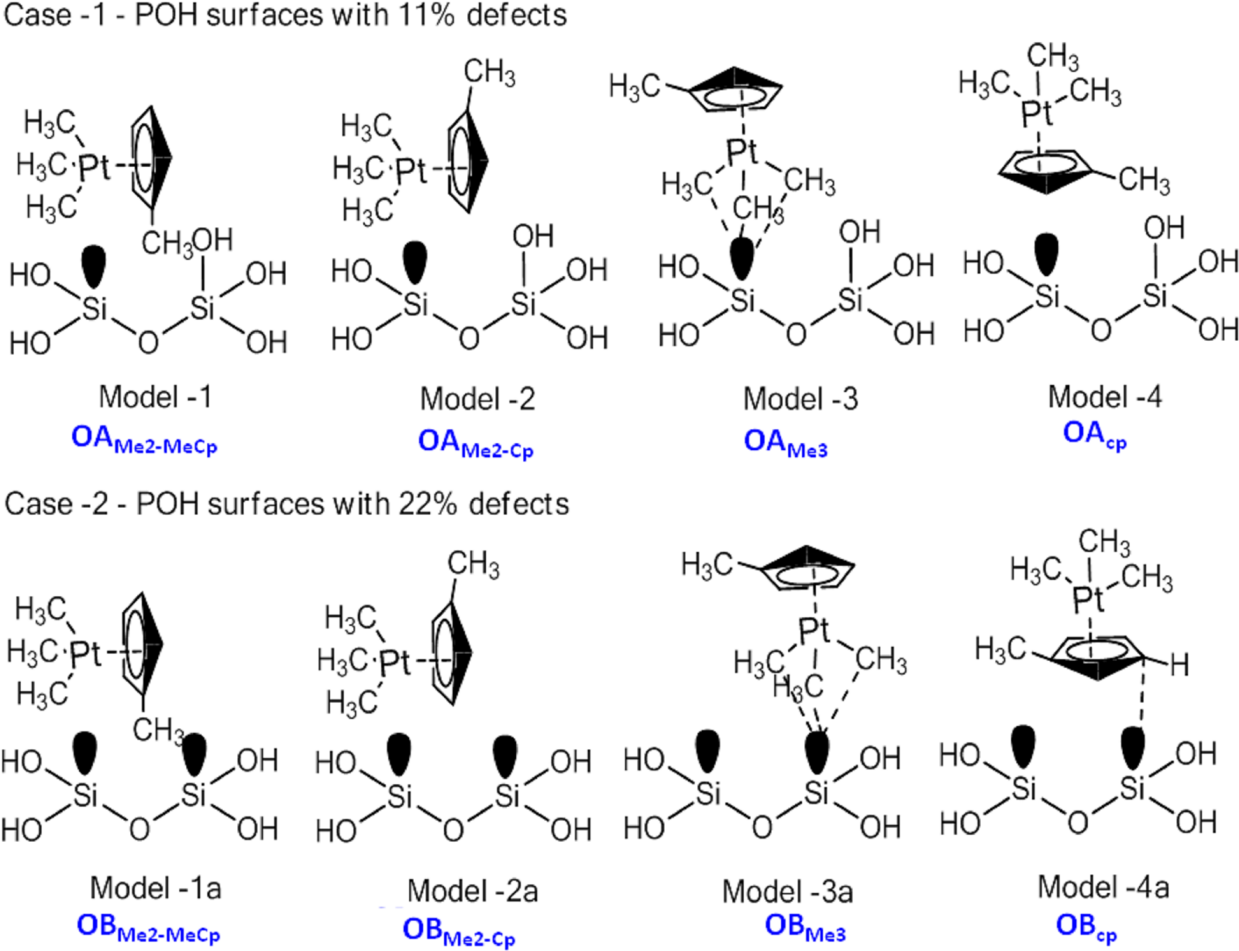
Schematic representation of the initial orientations of (C_5_H_4_CH_3_)Pt(CH_3_)_3_ on 11% (Model-1 to Model-4, upper panel) and 22% (Model-1a to Model-4a, lower panel) partially dehydroxylated surfaces.

**Figure 2:**
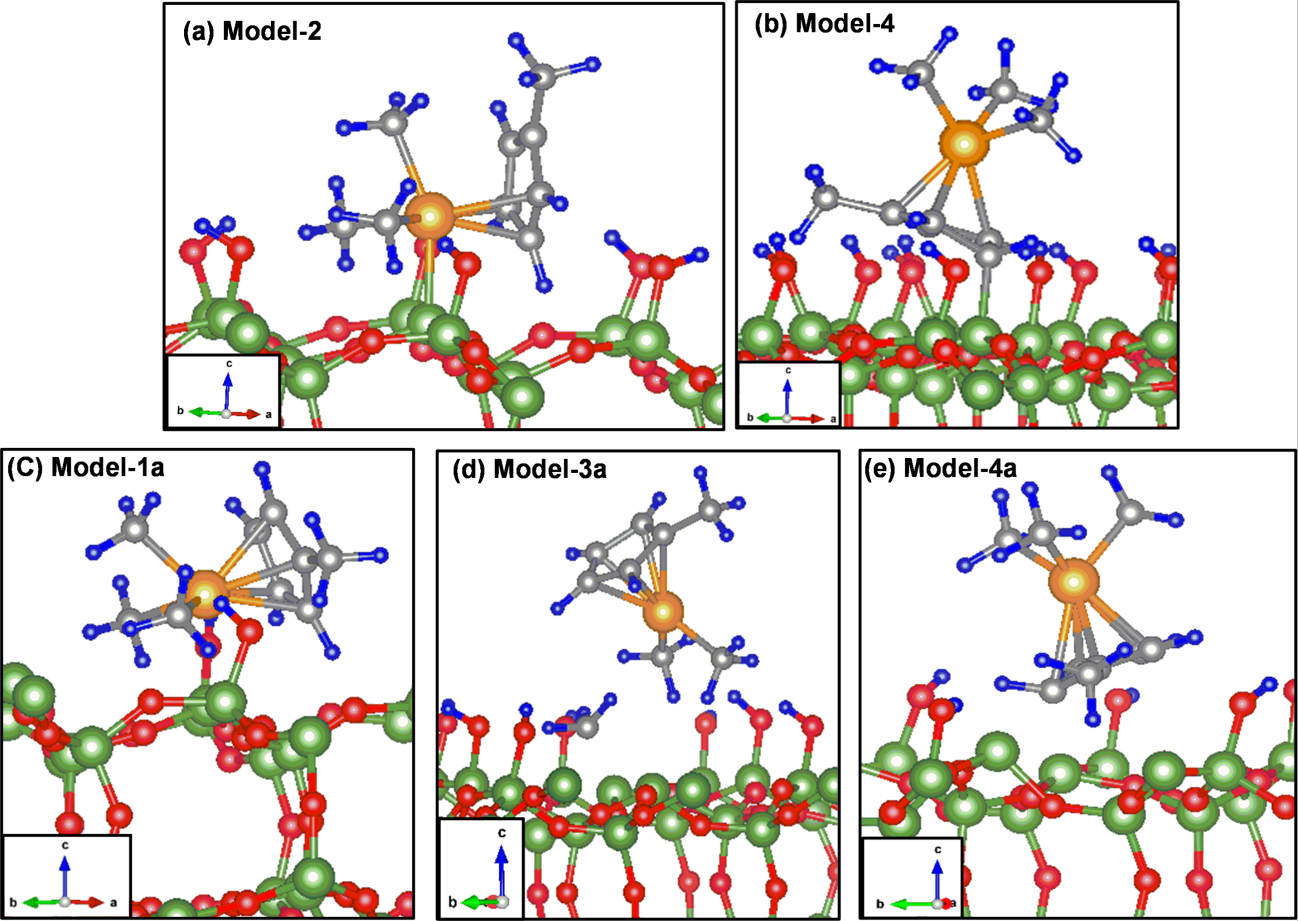
Relaxed structures of (C_5_H_4_CH_3_)Pt(CH_3_)_3_ on the (a, b) 11% and (c–e) 22% partially dehydroxylated surfaces. The labels correspond to the initial configurations shown in Figure 1. In the most stable configuration for the 22% case (panel d), one of the three methyl groups bonded to Pt dissociates spontaneously during relaxation. Color code: green – Si, red – O, orange – Pt, gray – C and blue – H throughout this manuscript. To better display the changes occurring the system in 2D view, the snapshots have slightly different orientations in the *ab*-plane (note the coordinate systems).

In a similar way, the bonding of (C_5_H_4_CH_3_)Pt(CH_3_)_3_ precursor molecules on partially hydroxylated SiO_2_ surfaces were investigated. The initial configurations of (C_5_H_4_CH_3_)Pt(CH_3_)_3_ considered for simulations on the partially hydroxylated surfaces are shown schematically in Figure 1. We simulate the precursor adsorption on two different partially hydroxylated surface models, that differ in the number of available active sites on the surfaces; 11% (Figure 1, upper panel) and 22% (Figure 1, lower panel). On these surfaces, two sets of (C_5_H_4_CH_3_)Pt(CH_3_)_3_ orientations were considered: (1) reclining orientations (Model-1/1a and Model-2/2a), that differ in the orientation of the methylcyclopentadienyl ring of (C_5_H_4_CH_3_)Pt(CH_3_)_3_, and (2) upright configurations (Model-3/3a and Model-4/4a) in which either three of the methyl groups bonded to Pt or the centroid of the methylcyclopentadienyl ring bonds to the substrate. These initial configurations are allowed to relax without any constraints. The configurations where (C_5_H_4_CH_3_)Pt(CH_3_)_3_ is placed on the bridging sites move spontaneously to on-top sites during geometry relaxation and hence are not discussed further. The calculated adsorption energies (*E*_A_) are summarized in Table 1 and the configurations are summarized in Figure 2.

**Table 1:** Adsorption energies of (C_5_H_4_CH_3_)Pt(CH_3_)_3_ on partially hydroxylated SiO_2_ surfaces with 11 and 22% defects. The models listed correspond to the configurations in Figure 1. All values in eV/unit cell.

	SiO_2_ surfaces with	
	11% defects	22% defects

model-1/1a	−0.680	−2.321
model-2/2a	−1.443	−2.297
model-3/3a	−0.332	−2.769
model-4/4a	−1.458	−2.360

The calculated values of *E*_A_ of (C_5_H_4_CH_3_)Pt(CH_3_)_3_ on 11% partially dehydroxylated surfaces indicates that the most stable configurations are Model-2 (OA_Me2Cp_, *E_A_* = −1.443 eV) and Model-4 (OA_Cp_, *E_A_* = −1.458 eV) given in Figure 2a,b. The configurations are equally stable within the computational error. The configurations Model-1 (OA_Me2MeCp_, *E_A_* = −0.680 eV) and Model-3 (OA_Me3_ , *E_A_* = −0.332 eV) are less stable and are not considered for discussion. In Model-4, where the centroid of the methylcyclopentadienyl ring is oriented on top of a Si atom in the initial configuration, a spontaneous bond formation between one of the carbon atoms of the ring and the surface Si atom is observed during relaxation. There has been no evidence for any further dissociation.

Bonding of (C_5_H_4_CH_3_)Pt(CH_3_)_3_ has also been considered on the 22% partially dehydroxylated surfaces and the relaxed configurations are shown in Figure 2c–e. The calculated adsorption energies for the reclining configurations Model-1a (OB_Me2MeCp_), and Model-2a (OB_Me2Cp_) are −2.321 and −2.297 eV, respectively. We do not observe any spontaneous dissociation on these configurations. However, when the relaxations are started with Model-3a orientation (OB_Me3_), a stronger adsorption with *E*_A_ = −2.769 eV is observed. In this case, one of the three methyl groups that bonds directly to the Pt atom dissociates, as has been observed in experimental investigations [3,25–28]. The detached methyl group was found to bind to one of the surface active sites. However, this situation was not observed on 11% partially dehydroxylated surfaces indicating that the dissociation is assisted by the neighboring active site present on the 22% partially dehydroxylated surfaces. The next most stable configurations on the 22% partially dehydroxylated surface are obtained from Model-4a ((OB_Cp_) *E*_A_ = −2.360 eV).

A comparison of the calculated adsorption energies for the 11% and 22% cases indicates that the molecules are more stable on the latter. Furthermore, no dissociation on the 11% dehydroxylated surface was observed. These observations illustrate that the orientation of (C_5_H_4_CH_3_)Pt(CH_3_)_3_ on SiO_2_ surfaces, and the availability and location of active sites on the surface are crucial factors in dictating the dissociation of precursors and the growth mechanism of deposits.

### Dynamics of the (C_5_H_4_CH_3_)Pt(CH_3_)_3_ precursor on SiO_2_ substrates

**Figure 3:**
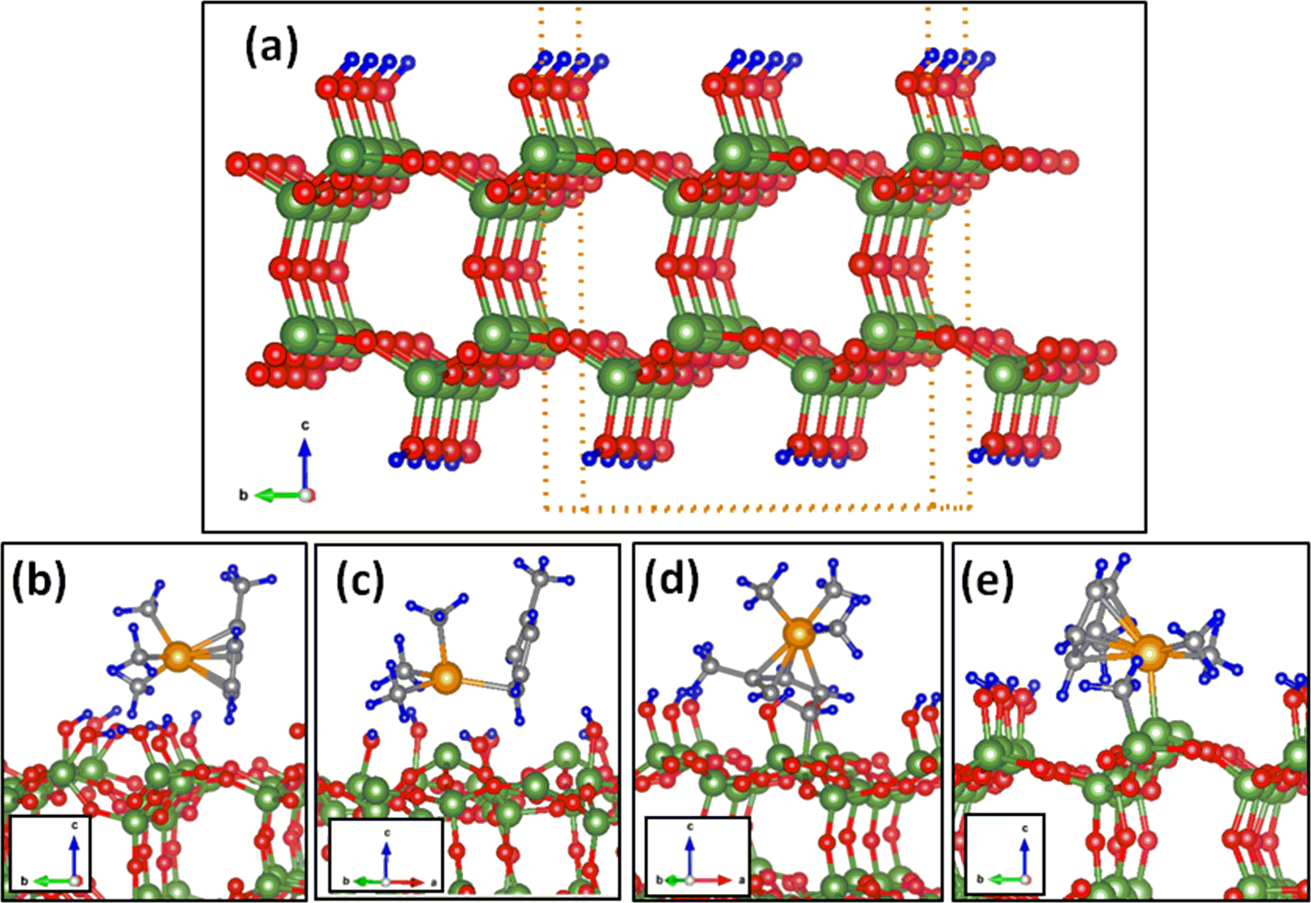
(a) Reduced supercell with the fully hydroxylated SiO_2_ considered for the molecular dynamics simulations. (b–e) Most stable configurations of (C_5_H_4_CH_3_)Pt(CH_3_)_3_ (b) on the fully hydroxylated surface, (c, d) on the 25% partially dehydroxlated, and (e) on the 50% partially dehydroxylated surfaces.

To gain further insight on the growth process of Pt deposits, dynamics of (C_5_H_4_CH_3_)Pt(CH_3_)_3_ on both fully and partially hydroxylated surfaces were simulated. Due to the high computational expense, however, a reduced 2 × 2 × 2 supercell of SiO_2_ (Figure 3a), was used. This reduced supercell provides a closer packing of precursor molecules (the nearest-neighbor distance between two Pt atoms in (C_5_H_4_CH_3_)Pt(CH_3_)_3_ is ca. 8 Å in the reduced cell compared to ca. 15 Å in the original supercell) and an enhanced concentration of surface hydroxyl vacancies is provided in the reduced cell on partially dehydroxylated surfaces (i.e., 25% and 50% compared to 11% and 22%). However, the environment around these sites is similar (i.e., the sites are isolated on the 25% partially dehydroxylated surface and located adjacent to each other on the 50% partially dehydroxylated surface).

Selected configurations on fully and partially hydroxylated SiO_2_ surfaces are computed using this reduced supercell and compared for consistency (Table 2). We observe a similar trend for the energetics and the order of stabilization of configurations as in the 3 × 3 × 4 cell. For example, the most stable configuration for (C_5_H_4_CH_3_)Pt(CH_3_)_3_ on the fully hydroxylated surfaces (Figure 3b) of both of these cells are similar. The (C_5_H_4_CH_3_)Pt(CH_3_)_3_ adsorption on 3 × 3 × 4 fully hydroxylated surface slabs has the most stable configuration O_Me2MeCp_ (*E*_A_ = −0.650 eV) followed by O_Me2Cp_ (*E*_A_ = −0.596 eV) [14]. On a 2 × 2 × 2 supercell, the most stable configuration is O_Me2MeCp_ (*E*_A_ = −1.02 eV) followed by O_Me2Cp_ (*E*_A_ = −0.845 eV). Analogously, on a 3 × 3 × 4 partially hydroxylated surface, the most stable configuration is Model-4 (*E*_A_ = −1.458 eV) followed by Model-2 (*E*_A_ = −1.443 eV) and a similar trend is observed with the reduced supercell, i.e., Model-4 (*E*_A_ = −1.647 eV) followed by Model-2 (*E*_A_ = −1.494 eV). Neverthless, the calculated values of *E*_A_ when using the reduced supercell are higher than those when using the 3 × 3 × 4 slabs, owing to the enhanced concentration of the hydroxyl groups and defect sites, and the closer packing of the molecules. On the 50% dehydroxylated cells, where two surface active sites are located adjacent to each other, one of the methyl groups, which was originally bonded to Pt, fragments and bonds to the adjacent vacant site, as observed in the 3 × 3 × 4 cell (see Figure 2d and Figure 3e). These DFT relaxed structures (as shown in Figure 3b–e) are considered as starting point for molecular dynamics simulations.

**Table 2:** Comparison of (C_5_H_4_CH_3_)Pt(CH_3_)_3_ adsorption energies on the 3 × 3 × 4 supercell and the reduced 2 × 2 × 2 supercell. For the fully hydroxylated surfaces, the values are taken from [14]. Model-2 and Model-4 are the stable configurations of (C_5_H_4_CH_3_)Pt(CH_3_)_3_ on partially hydroxylated surfaces. All values in eV/ unit cell.

	supercell	
	3 × 3 × 4	2 × 2 × 2

fully hydroxylated (O_Me2MeCp_)	−0.650	−1.02
fully hydroxylated (O_Me2Cp_)	−0.596	−0.845
Model-2	−1.443	−1.494
Model-4	−1.458	−1.647

**Figure 4:**
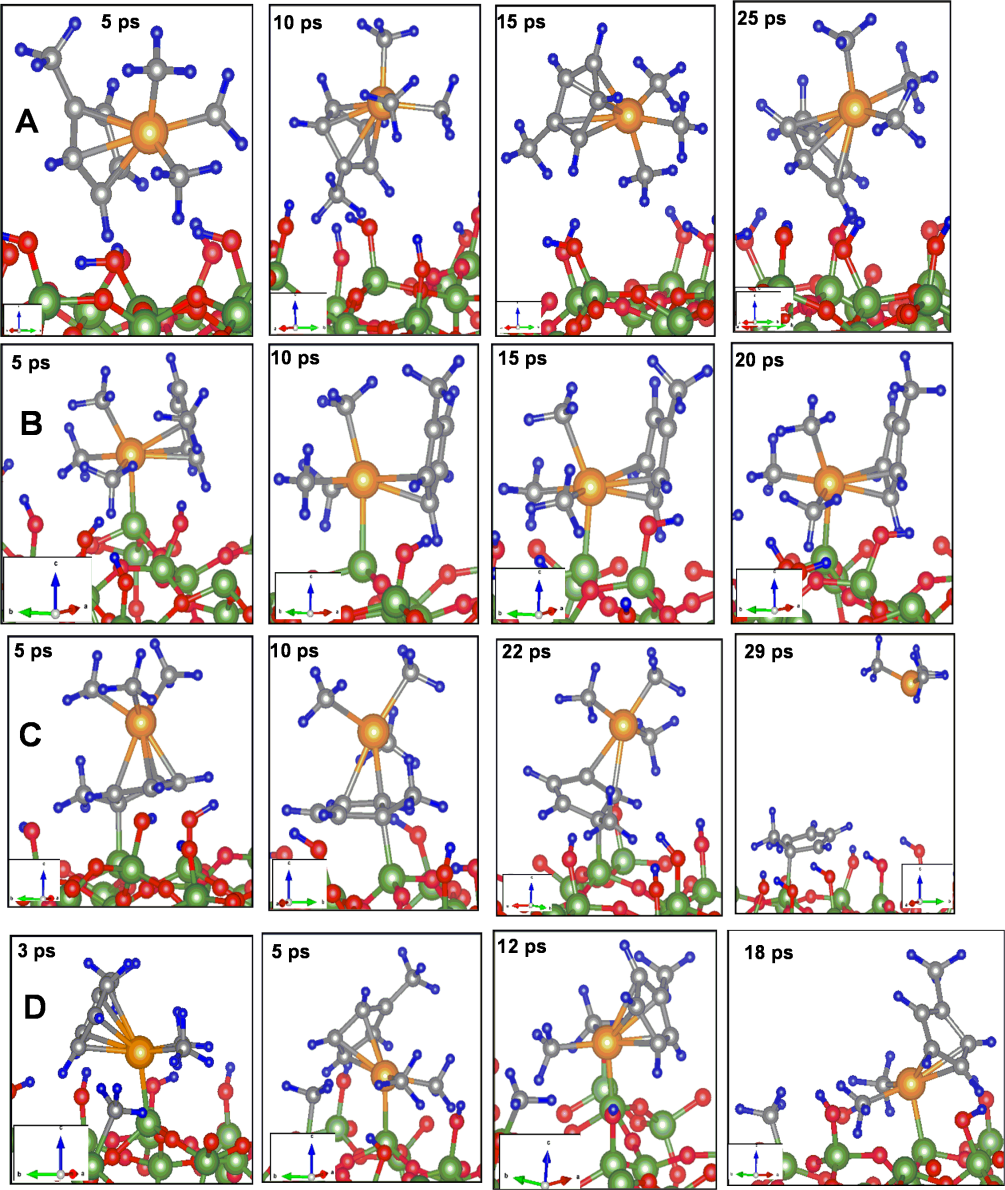
The configurations of (C_5_H_4_CH_3_)Pt(CH_3_)_3_ obtained from molecular dynamics simulations (20 ps) on (a) fully hydroxylated surfaces, (b)-(c) 25% and (d) 50% partially dehydroxylated surfaces

The trajectory of the MD simulations for the four considered cases are shown in Figure 4. The analysis of the trajectory indicates that on the fully hydroxylated surfaces (Figure 4a) the molecule exhibits significant changes in orientation and a drift similar as we found for carbonyl precursors [11]. The Pt–Cp ring and Pt–methyl bonds fluctuate with a maximum of 5% and 1%, respectively. Apart from this, in the simulation window, we do not observe any further indications of the dissociation of the precursor molecules. The trajectory of MD simulations and arising configurations of (C_5_H_4_CH_3_)Pt(CH_3_)_3_ on the (25% and 50%) partially dehydroxylated SiO_2_ surfaces in MD simulations are shown in Figure 4b–d. When simulation is started with the configuration shown in Figure 3b, a significant bond weakening of Pt–Cp ring and Pt–methyl is observed (Figure 4b). Quantitatively, the respective bond fluctuations computed for Pt–Cp ring and Pt–methyl bonds were 11% and 9%, respectively. However, when MD simulations are performed with Figure 3d as starting configuration, where a part of the methylcyclopentadienyl ring is bonded to the surface Si atom during the simulations, the bonding of that part with the Pt(CH_3_)_3_ part of the precursor is weakened. When simulations are extended further, the Pt(CH_3_)_3_ part detaches and moves to the vacuum leaving the methylcyclopentadienyl ring bonded to the substrate (see Figure 4c). On the 50% partially dehydroxylated surface, (Figure 4d), no further association or dissociation of methyl groups or detachment of methylcyclopentadienyl rings are observed.

These results illustrate that the (C_5_H_4_CH_3_)Pt(CH_3_)_3_ precursor molecules exhibit a tendency to fragment upon their interaction with the partially hydroxylated sites and a drifting without fragmentation on the fully hydroxylated surfaces. On the partially hydroxylated surfaces, the dissociation begins either with the release of one of the methyl groups bonded directly to Pt or the detachment of the methylcyclopentadienyl ring. This indicates that surface active sites are necessary for the fragmentation of precursor molecules and this is because of the electron density located on the Si atoms, which is crucial for bonding with the precursor molecule and further fragmentation [9–10]. The 25% and 50% partially dehydroxylated surfaces represent electron beam pretreated surfaces and therefore the pretreatment of the substrate with the electron beam helps in favoring dissociation of the precursor molecule and efficient deposition. Since during the MD simulations we do not observe the dissociation of the methylcyclopentadienyl ring or the release of methyl groups from the surface Si sites, it is expected that they might block the active sites from the approach of incoming precursor molecules.

### Pathways for (C_5_H_4_CH_3_)Pt(CH_3_)_3_ precursor fragmentation

In order to further understand the nature of the interaction of (C_5_H_4_CH_3_)Pt(CH_3_)_3_ with partially hydroxylated SiO_2_ surfaces, the barriers for adsorption and fragmentation of these molecules to the partially hydroxylated groups were simulated. For this purpose, a representative simple cluster model Si(OH)_3_ is considered. Although this does not account for the complete surface model, it is a good approximation to evaluate the reaction barriers for different reactions observed through the MD simulations. In the literature, such a simple hydroxylated M(OH)*_x_* model has been used as a representative model to investigate deposition reactions on Al [29], Hf [13] and Si [30–32]. Also, the energies reported in this section are computed at the PM6 level of theory, and a relative comparison should enable reasonable understanding of the possible fragmentation pathways qualitatively. In a recent investigation, thickness-controlled site-selective Pt deposits were obtained by direct atomic layer deposition (ALD) in which ALD was performed on EBID patterned substrates. In this ALD process, an O_2_ pulse is used to obtain better nucleation, even though the qualitative understanding of the role of O_2_ remains uncertain. Therefore, in this section we consider the involvement of O_2_ in the pathways at different stages of the reaction and calculate the energetics to compare and elucidate the role of O_2_ [33].

Our simulations of the adsorption and dynamics of (C_5_H_4_CH_3_)Pt(CH_3_)_3_ on SiO_2_ surfaces show that the dissociation begins with either the detachment of methyl groups or of the methylcyclopentadienyl ring. In the cluster model, the interaction of (C_5_H_4_CH_3_)Pt(CH_3_)_3_ with the surface Si atoms, has a barrier of 0.140 eV, when (C_5_H_4_CH_3_)Pt(CH_3_)_3_ interacts through the methylcyclopentadienyl ring. Removal of the methylcyclopentadienyl ring from (C_5_H_4_CH_3_)Pt(CH_3_)_3_ has an activation energy of +0.771 eV, which leaves the Pt(CH_3_)_3_ part of the precursor bound to the substrate. The formation of Pt(CH_3_)_3_ in vacuum is observed in our molecular dynamics simulations. However, neither its dissociation into vacuum nor its adsorption back to the surface Si sites have been observed. Therefore, for analyzing the dissociation channels of Pt(CH_3_)_3_ we reasonably approximate our starting configuration as Pt(CH_3_)_3_ bonded to the surface Si site as shown in Scheme 1. Our adsorption studies reveal that for the elimination of methyl groups two surface active sites are necessary, and the model used here limits the computation of the reaction barriers to such cases.

**Figure 5:**
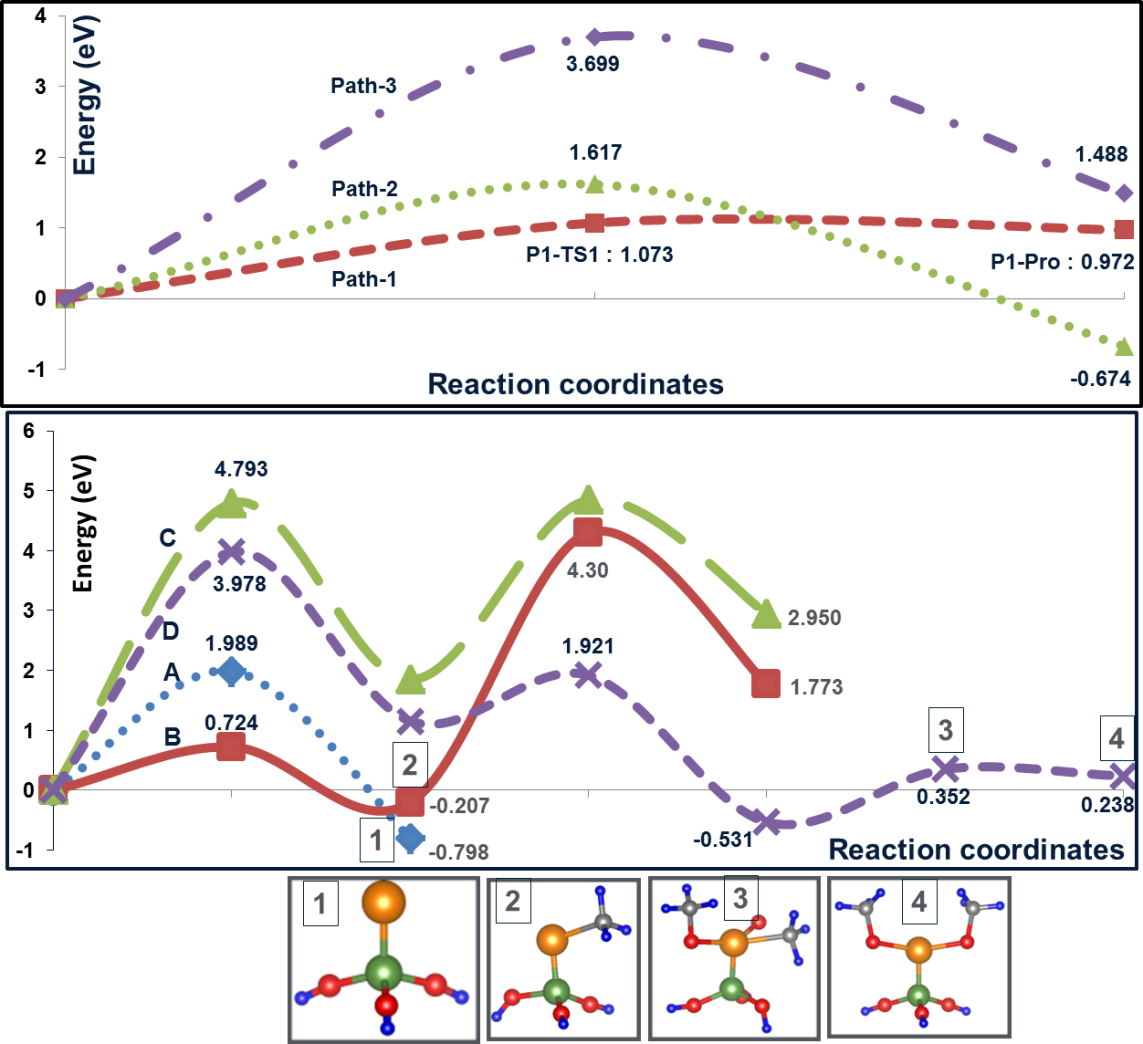
Energetics for different pathways (Path 1–3 and Path A–D) for the dissociation of precursor molecules.

Possible pathways by which Pt(CH_3_)_3_ gets dissociated on the model cluster, in the presence and absence of O_2_ , are derived and shown in Scheme 1. From Pt(CH_3_)_3_, the release of one methyl group (P1TS1) forming Pt(CH_3_)_2_ has an activation energy of about +1.073 eV compared to +1.617 eV for the release of ethane (P2TS1). The O_2_ -assisted elimination of the first methyl group (P3TS1) from Pt(CH_3_)_3_ has a barrier of +3.699 eV. Therefore, the release of the first methyl group is expected to proceed through P1TS1, leaving Pt(CH_3_)_2_ (P1-PRO-1) on the Si surface. The removal of the second and the third methyl groups from Pt(CH_3_)_2_ can occur through a number of ways (Path A to Path D in Scheme 1 and Figure 5). Ethane elimination, which leads to a Pt atom (PA-TS1) bonded to the Si surface has a barrier of about +1.989 eV. This reaction is exothermic by −0.798 eV. The first step (PBTS-1) in Path B leading to a second methyl elimination has a barrier of +0.724 eV and occurs in a similar fashion as the first methyl removal. This reaction resulting in species PBINT-1 is exothermic by −0.207 eV. Comparing the pathways considered and the computed activation energies as shown in Figure 5, the third methyl elimination in presence of oxygen, and the subsequent formation of Pt-oxy species (Path-B, C, D), have a high activation barrier.

The formed Pt and Pt–oxygen species might agglomerate and form Pt deposits and the role of O_2_ on this process has not been explored in this investigation. Some experimental studies are available and possible mechanisms were proposed [34]. Simulations in this study were performed at the PM6 level, with no corrections for weak interactions and at *T* = 0 K. Taking into account the temperature of most processes, which ranges between 200 and 400 °C, some of the high-barrier pathways might also be operative. Also, the role of secondary electrons and the point defects that might occur as a result of electron impingement on the surface has not been explored. This study therefore provides a preliminary understanding of how the (C_5_H_4_CH_3_)Pt(CH_3_)_3_ molecule can dissociate on substrates that qualitatively represent electron beam pretreated surfaces.

## Conclusion

We have performed theoretical simulations on the dynamics and fragmentation mechanisms of (C_5_H_4_CH_3_)Pt(CH_3_)_3_ on fully and partially hydroxylated SiO_2_ surfaces. Our results provide clues for the most stable configurations of (C_5_H_4_CH_3_)Pt(CH_3_)_3_ on SiO_2_ surfaces and their dynamical behavior. These results illustrate that the fragmentation of the precursor molecule begins with either the detachment of either the methylcyclopentadienyl ring or the methyl group. Detached methylcyclopentadienyl rings and the dissociated methyl groups block the surface active sites and might be the source of organic contamination. Since the composition of the obtained deposits dictate the conductance behavior, it can be speculated that a design of suitable precursors for electron beam induced deposition might be more efficient than the use of traditional ALD precursors. With our reaction modeling studies, possible pathways by which the precursor molecule can fragment on SiO_2_ surfaces were also explored.

## Acknowledgments

The authors gratefully acknowledge financial support by the Beilstein-Institut, Frankfurt/Main, Germany, and the EU COST action CELINA. The generous allotment of computer time by CSC-Frankfurt and LOEWE-CSC is gratefully acknowledged.
